# SEX-SPECIFIC ALTERATIONS IN CIRCULATING LIPIDOME DURING MOUSE AGING

**DOI:** 10.1093/geroni/igae098.1869

**Published:** 2024-12-31

**Authors:** Ziying Xu, Anindita Bhattacharjee, Xianlin Han

**Affiliations:** Barshop Institute, San Antonio, Texas, United States; Barshop Institute, San Antonio, Texas, United States; Barshop Institute, San Antonio, Texas, United States

## Abstract

Lipids serve as pivotal elements in biological systems, fulfilling diverse roles ranging from cellular structure components to signaling molecules and energy reserves. They encompass a variety of classes including structural membrane lipids (e.g., cholesterol, phosphatidylcholine, phosphatidylethanolamine), energy-storing lipids (e.g., triglycerides, fatty acids), and bioactive lipids (e.g., lyso-phospholipids, ceramides). Increasing evidence shows associations between lipids and age-related diseases (e.g., atherosclerosis, cancer, diabetes, and neurodegenerative diseases), indicating lipid metabolism is involved with health and longevity. In this study, we applied multi-dimensional mass spectrometry-based shotgun lipidomics to quantitatively measure the plasma lipidome alterations across different ages in C57BL/6 mice. We systematically collected plasma samples from both sexes across 7 adult-to-old-age timepoints (8 to 32 months, in 4-month increments, with 8-10 mice per sex at each age). Our data revealed a sex-dependent regulation within the circulating lipidome in which males demonstrated higher concentrations across nearly all lipid classes at each timepoint compared to females. Notably, with advanced ages, males exhibited a decline in major phospholipids and an accumulation of cholesteryl ester—suggestive of an age-related shift in lipoprotein composition. An age-associated increase in phosphatidylserine, previously linked to apoptotic signaling, was exclusive to males. Conversely, females showed a consistent plasma lipid profile throughout their lifespan. The only shared change between sexes is plasmalogen decreasing with age, which could imply increasing oxidative stress. Our findings illuminate sex-specific regulatory mechanisms in lipid metabolism during aging in mice, with potential implications for understanding sex differences in the aging process.

